# Multiple Origins of Elytral Reticulation Modifications in the West Palearctic *Agabus bipustulatus* Complex (Coleoptera, Dytiscidae)

**DOI:** 10.1371/journal.pone.0009034

**Published:** 2010-02-03

**Authors:** Marcus K. Drotz, Tomas Brodin, Anders N. Nilsson

**Affiliations:** 1 Lake Vänern Museum of Natural and Cultural History, Lidköping, Sweden; 2 Department of Ecology and Environmental Science, Umeå University, Umeå, Sweden; American Museum of Natural History, United States of America

## Abstract

The *Agabus bipustulatus* complex includes one of Europe's most widely distributed and common diving beetles. This complex, which is known for its large morphological variation, has a complex demographic and altitudinal variation in elytral reticulation. The various depth of the reticulation imprint, both in smaller and larger meshes, results in both mat and shiny individuals, as well as intermediate forms. The West Palearctic lowland is inhabited by a sexually dimorphic form, with shiny males and mat females. In mountain regions, shiny individuals of both sexes are found intermixed with mat individuals or in pure populations in central and southern areas, whereas pure populations of mat individuals are exclusively found in the northern region at high altitude. Sexual selection is proposed as a driving force in shaping this variation. However, the occurrence of different types of reticulation in both sexes and disjunct geographical distribution patterns suggest an additional function of the reticulation. Here we investigate the phylogeographical history, genetic structure and reticulation variation of several named forms within the *Agabus bipustulatus* complex including *A. nevadensis*. The molecular analyses recognised several well-supported clades within the complex. Several of the named forms had two or more independent origins. Few south European populations were uniform in reticulation patterns, and the males were found to display large variation. Reticulation diversity and population genetic variability were clearly correlated to altitude, but no genetic differences were detected among populations with mixed or homogenous forms. Observed reduction in secondary reticulation in female and increased variance in male at high altitude in South Europe may be explained by the occurrence of an additional selective force, beside sexual selection. The combined effect of these selective processes is here demonstrated in an extreme case to generate isolation barriers between populations at high altitudes. Here we discuss this selective force in relation to thermal selection.

## Introduction

The elytral surface of most diving beetles is covered by a reticulation pattern impressed on the elytra, often consisting of small and large meshes – the primary and secondary reticulation [1]–[3]. The primary reticulation consists of a more or less regular fine meshwork, whereas the secondary reticulation is coarser with larger meshes of a more irregular, often oblong, shape. Sexual dimorphism is commonly found, especially in geographically disjunct areas at high altitude or gradually between longitudinal areas in e.g. the West Palaearctic region. Traditionally, these reticulation patterns have taxonomically been recognised as a set of characters used to identify and separate species, subspecies and varieties within several species complexes [1], [4]–[8]. However, little effort has been taken to understand the evolutionary significance of the sexual and altitudinal variation in elytral microsculpture and reticulation patterns occurring within a single species complex. This view changed when Bergsten and co-workers [9] nicely demonstrated an ongoing arms-race including female elytral sculpture and the number and size of adhesive male pro- and mesotarsal setae in several dytiscine genera. Miller [10] and subsequent studies [11]–[14] strengthened these observations and argued with support from the general model of sexual conflict [15]–[16] that the first step of a female response to increased mating cost is the occurrence of an increased female reticulation compared to males with tarsal modifications.

Accordingly, these findings provide an explanation of the sexual dimorphism and increased modification of female reticulation in dytiscids. However, the observation that both males and females in some water beetles display a large variation in primary and secondary reticulation indicates that the microsculpture most likely has evolved for some other reason. Moreover, the occurrence of similar reticulation patterns at high altitude in different mountain ranges across large geographical areas also indicates the presence of an additional selective agent or common evolutionary history [17]–[19]. Sexual selection could therefore be a selective mechanism that enhances or contributes secondary to the diversification of reticulation patterns at high altitude in some species [20]–[22].

Clearly, several underlying evolutionary factors need to be considered in order to understand why different aberrant reticulation patterns, such as a more strongly impressed male secondary reticulation and more shiny females, occur at high altitude in contrast to the more homogeneous reticulation pattern normally observed at lower altitudes [19], [23]–[24]. Here we will focus on the phylogeographic history of different reticulation patterns and genetic differences between populations consisting of various reticulation forms in relation to altitude observed in the *Agabus bipustulatus* (Linnaeus, 1767) species complex.

**Figure 1:**
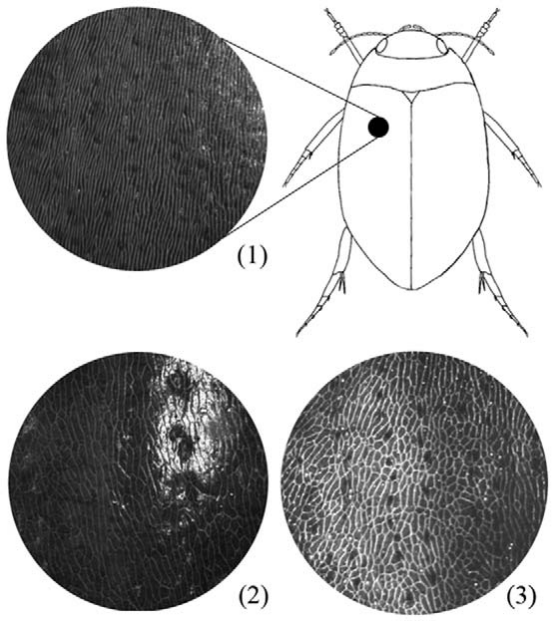
*Agabus bipustulatus* secondary elytral reticulation patterns. (1) Reticulation classified as type A and found within the topotypic male *bipustulatus* form, (2) as type B found within *kiesenwetterii*, (3) as type G found within the *pyrenaeus* form. Reticulation classes are coded as in table 2.

This species complex is known to display a pronounced individual shape variation in combination with an elytral reticulation that varies both between and within sexes, especially at higher altitudes [1]–[8], [13], [25]–[26]. The pronounced morphological, geographical and altitudinal variation is reflected taxonomically in the 23 available junior synonyms of uncertain taxonomic status [8]. The montane form named *Agabus solieri* Aubé, 1837 has a body-shape, especially seen on the pronotum, which is more slender than in the widely distributed topotypic lowland form *bipustulatus* [25], [27]. Most of the other named forms are montane, and found in populations imbedded in areas dominated by the *solieri*-like form. Main differences between the forms, which taxonomically often have been dealt with as subspecies or varities of either *bipustulatus* or *solieri*, are the shininess of the elytra and different patterns in their secondary reticulation (Fig. 1). In particular, the *kiesenwetterii* form stands out since both sexes have wider meshes in the secondary reticulation, and the elytra are more shining relative to the regular *solieri* form. The geographical distribution of these two forms is interesting since the *kiesenwetterii* form commonly is found at high altitude in central and south Europe imbedded in populations dominated by the duller *solieri* form, whereas *solieri* is the only form occurring in North Europe [1]. These forms are suggested to be subjected to sexual selection which is seen in populations where males evolve wider tarsi and attached suckers in the presence of modified reticulated females [13]. In other known forms, only the male displays reticulation variation, whereas the females are homogenous for a given pattern.

The Iberian endemic *Agabus nevadensis* Hå. Lindberg, 1939 is here of interest due to its geographically restricted high-altitude occurrence in the Sierra Nevada and morphological similarity to *A. bipustulatus*. This local endemic has a reticulation pattern identical to that of the *kiesenwetterii* form in both sexes [28]. Its species status has been questioned and it is commonly included within the *A. bipustulatus*-complex [7], [26].

## Methods

### Sample Collection

**Figure 2:**
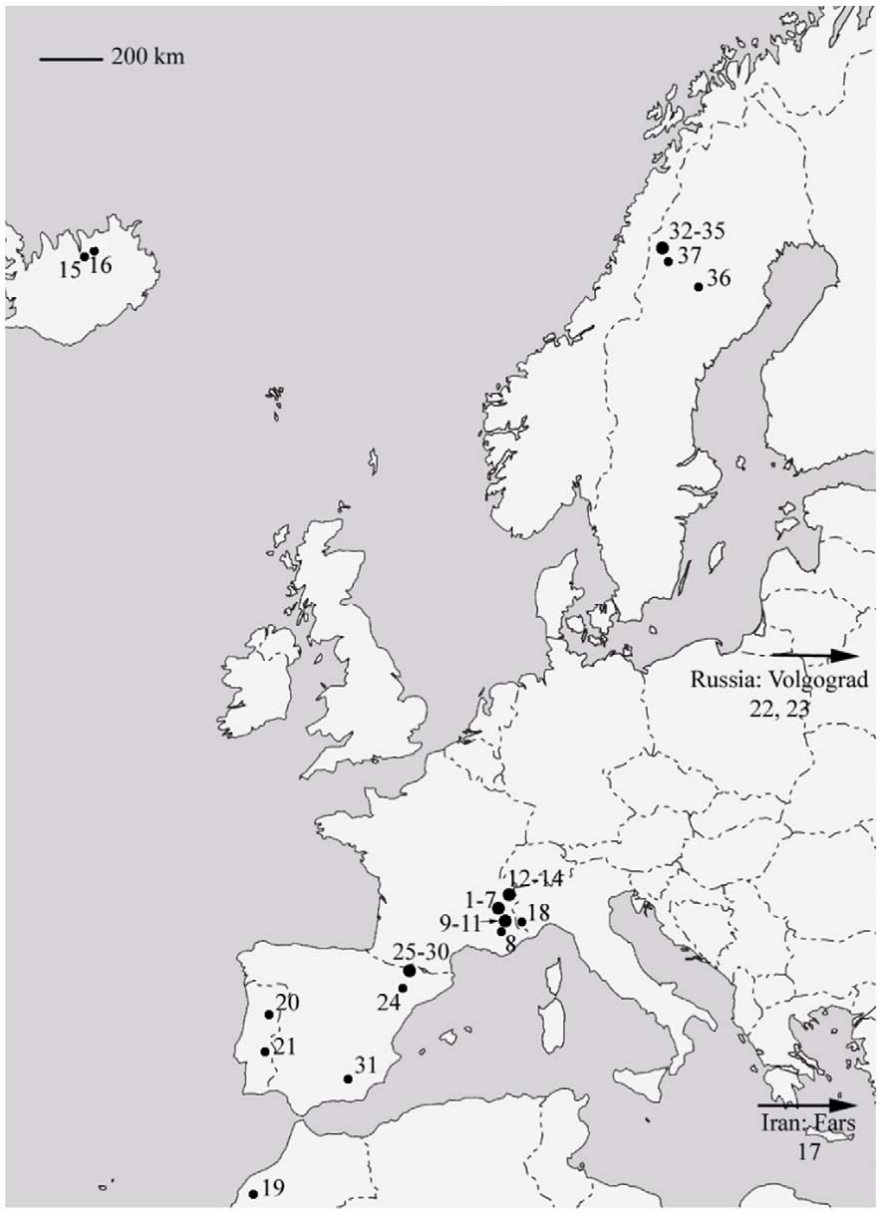
*Agabus bipustulatus* collecting sites. Map codes and corresponding locality codes are given in table 1.

A total of 717 specimens representing six of the named forms of *A. bipustulatus* were collected from 37 sample sites across its West Palearctic distribution area in order to capture as large genetic and morphological variation as possible (Table 1, Fig. 2). All specimens used in the population genetic analysis were also included in the morphological analysis of the secondary reticulation.

**Table 1:** Sampled species and collection sites along with their respective locality code.

Species	Country	Locality	Locality code	Collector	Map code
*A. affinis*	Sweden	Västerbotten; Umeå, Nydalasjön	Aff1	AN	
*A. bipustulatus*	France	**Grenoble; Huez, NW of Lac Blanc**	**BipFra1**	MD	**1**
		**Grenoble; Huez, N of Lac Blanc**	**BipFra2**	MD	**2**
		**Grenoble; Chamrouse, la Botte**	**BipFra3**	MD	**3**
		**Grenoble; Chamrous, Ch^et^ de I**'**Oursiere**	**BipFra4**	MD	**4**
		**Grenoble; Séchilienne, Pas de I**'**Envious**	**BipFra5**	MD	**5**
		**Grenoble; Séchilienne, Mont-Sec**	**BipFra6**	MD	**6**
		**Grenoble; Séchilienne, le Couvênt**	**BipFra7**	MD	**7**
		**Guillestre; Col de Vars**	**BipFra8**	MD	**8**
		**Guillestre; Pic de Balart, Lac de Néal**	**BipFra9**	MD	**9**
		**Guillestre; Bois Noir, Lac du lauzet sup.**	**BipFra10**	MD	**10**
		**Guillestre; Bois Noir, Lac du lauzet inf.**	**BipFra11**	MD	**11**
		**Montcenisio; les Coulours, Lacs Giaset**	**BipFra12**	MD	**12**
		**Montcenisio; les Coulours, le Lac Blance**	**BipFra13**	MD	**13**
		**Montcenisio; les Coulours, Savins Runes**	**BipFra14**	MD	**14**
	Iceland	Eyjafjörður sýsla; Akureyri airport	BipIce1	SE	15
		Eyjafjörður sýsla; South of Akureyri	BipIce2	SE	16
	Iran	Fars; Shiraz	BipIra1	SH	17
	Italy	**Cuneo; Monte Mongioie, Brignola**	**BipIta1**	MD	**18**
	Morocco	Tafraoute; Oued Åit Baha Ait Iftene	BipMor1	IR	19
	Portugal	Guarda; Sa da Estrele	BipPor1	IR	20
		Portalegre, Serra de São Mamede	BipPor2	IR	21
	Russia	Volgograd Oblast; Volgograd	BipRus1	AN	22
		Volgograd Oblast; Volgograd	BipRus2	AN	23
	Spain	Tortosa; El Pinell de Brai	BipEsp1	MD	24
		Catalonia, Coll de Perves	BipEsp2	MD	25
		**Catalonia; Vielha, Port de la Bonaigua**	**BipEsp3**	MD	**26**
		**Catalonia; Vielha, Lacs de Colomers**	**BipEsp4**	MD	**27**
		**Catalonia; Vall de Boí, N of Estany Nere**	**BipEsp5**	MD	**28**
		**Catalonia; Vall de Boí, Estany de Durro**	**BipEsp6**	MD	**29**
		**Catalonia; Vall de Boí, Estany del Bergús**	**BipEsp7**	MD	**30**
		Granada; Sierra Nevada, Hotel del Buque	BipEsp8	CSC	31
	Sweden	Lycksele lappmark; Tärna, Atoklinten	BipSwe1	MD	32
		Lycksele lappmark; Tärna, Djuptjärn	BipSwe2	MD	33
		Lycksele lappmark; Tärna, Gröndal	BipSwe3	MD	34
		Lycksele lappmark; Tärna, Stintbäcken	BipSwe4	MD	35
		Lycksele lappmark; Lycksele, Näslandsmyren	BipSwe5	MD	36
		Lycksele lappmark; Tärna, Kråkberget	BipSwe6	MD	37
*A. guttatus*	Sweden	Ångermanland; Nordmaling, Mullsjö	Gutt1	AN	
*A. melanarius*	Sweden	Lycksele lappmark; Tärna, Atoklinten	Mel1	MD	
		Ångermanland; Nordmaling, Hummelholm	Mel2	MD	
*A. nebulosus*	Sweden	Skåne; Potten	Neb1	BA	
		Skåne; Revinge by	Neb2	BA	
*A. nevadensis*	Spain	Granada; Sierra Nevada, Laguna de Rio seco	Nev1	MD	
		Granada; Sierra Nevada, Laguna de la Caldera	Nev2	MD	
*A. tristis*	Canada	Alberta; Hinton	Tri1	JB	
		Alberta; Sundre	Tri2	JB	
		Alberta; Sundre	Tri3	JB	
*A. wollastoni*	Portugal	Madeira; Pico do Arierio	Woll1	MD	
		Madeira; Rabacal	Woll2	MD	

Collectors of the populations/specimens are: Anders Nilsson (AN), Bertil Andrén (BA), Carmen E. Sainz Cantero (CSC), Ignacio Ribera (IR), Johannes Bergsten (JB), Marcus K. Drotz (MD), Stefan Ericsson (SE), and Shidi O. Hosseinie (SH). Map codes are given for *A. bipustulatus* collection sites, which are shown in figure 2. Collection sites in bold were used both in the morphological and population genetic studies.

In France, 14 *A. bipustulatus* populations were sampled near Grenoble, Guillestre, and Montcenisio. The type locality of *A. solieri* lies within the Grenoble region, and Guignot [7] described *A. solieri* var. *falcozi* from Montcenisio. Seidlitz [29] described *A. solieri* var. *kiesenwetterii* from Illyria, Piemonte and the Pyrenees, and it is according to Guignot [7] now also found it at high altitudes in the French Alps. One Italian locality, from which *A. bipustulatus dolomitanus* Scholz, 1935 is known, was visited [26]. Five populations were sampled in the Spanish Pyreenes near Vall de Boí in Catalunya, including the water system from which *A. solieri pyrenaeus* was described [30]. A total of 413 *A. nevadensis* specimens from eight populations were sampled near its type locality in central Sierra Nevada [28] (Table 1, Fig. 2).

To analyse the evolutionary background of different reticulation forms we utilised two *A. nevadensis* specimens and 30 *A. bipustulatus* specimens representing the reticulation pattern of 16 *bipustulatus*, two *kiesenwetterii*, three *falcozi*, one *dolomitanus*, one *pyrenaeus* and seven *solieri* forms. Individual specimens were collected to represent different secondary reticulation forms from different geographical regions with the aim to maximise the haplotypic (*mtDNA*) variation. The optimal outgroup taxa for the *A. tristis* species group is *A. bipustulatus* and *A. nevadensis*, according to molecular analyses of the Agabini, species from the *A. nebulosus* group [31]. Two *A. nebulosus* (Forster, 1771) specimens were therefore included, together with one *A. affinis* (Paykull, 1798) and one *A. guttatus* (Paykull, 1798). The former represents a basal clade of the subgenus *Gaurodytes*, whereas the latter belongs to the sister group of the *A. nebulosus* plus *A. tristis groups* [31]. Seven specimens of the *A. tristis* group not belonging to the *A. bipustulatus* complex were also included in the phylogenetic analysis (Table 1): representing the European Madeiran endemic *A. wollastoni* Sharp, 1882 the North Palearctic *A. melanarius* Aubé, 1837 and the Holarctic *A. tristis* Aubé, 1838.

Collected specimens that were used in the population genetic and phylogenetic analyses were transported alive in damp moss to the laboratory, where they were sexed and frozen at −70°C. Morphologically analysed specimens were stored in 96% ethanol in a refrigerator at 4°C.

### Reticulation Patterns

The reticulation patterns of the sampled forms are as follows; in North Europe, from the high mountains of Scandinavia to the lowland region below the Alps and the Pyrenees, the *A. bipustulatus* complex is represented by a sexually dimorphic form with a more or less constant elytral reticulation pattern. The males are shinier than the females and the secondary reticulation of both sexes consists of longitudinally stretched meshes. In the montane *kiesenwetterii* form both sexes have wide meshes in the secondary reticulation and the elytra are shinier than in topotypic *A. bipustulatus* from Sweden. Guignot [7] observed that *falcozi* males have wider secondary reticulation and are not as shining as those of *kiesenwetterii*, whereas the females are similar to the normal *bipustulatus* form. Only the males of *pyrenaeus* are shiny and have a more isomorphic secondary reticulation, and females have been described as similar to those of *bipustulatus* [30]. The latter information is not fully correct, however, and we will here demonstrate that the *pyrenaeus* females also display a similar variation as the males. The *dolomitanus* form is similar to *falcozi*, but it has the pronotal shape of lowland *bipustulatus* [32]. Franciscolo [32] also concluded, without any quantitative analysis, that the variation in secondary reticulation in *kiesenwetterii* and *dolomitanus* is so similar that they have to be conspecific.

Individual variation in the secondary elytral reticulation was classified after the total sample was screened for common patterns. Seven categories (A - G) ranging from tightly packed longitudinally elongated meshes to more or less isomorphic meshes were recognised (Fig. 1). Of these categories, the A, B, C and G patterns cover most of the total surface of the elytra (Table 2). The remaining categories D, E and F describe intermixed patterns of longitudinally elongated and isomorphic meshes. The A reticulation pattern is found in the bipustulatus and *solieri* forms. The B pattern is found in *kiesenwetterii*, *falcozi* and within the *dolomitanus* form. Within *pyrenaeus* we recognised three reticulation patterns: C, F and G.

**Table 2:** *Agabus bipustulatus* classification of secondary elytral reticulation after the total sample was screened for common patterns.

Code	Description
A	Longitudinally elongated meshes more or less straight, narrow and a majority are long. Isomorphic meshes can be observed at the elytral apex.
B	Longitudinally elongated meshes more or less straight, wide and a majority are long. Isomorphic meshes are observed commonly at the elytral apex and occasionally along the anterior parts of the elytra suture.
C	Longitudinally elongated meshes more or less straight, wide and a majority are short.
D	Longitudinally elongated meshes more or less straight, wide and a majority are long. Isomorphic meshes are observed from elytral apex to ½ or ¾ of its length.
E	Longitudinally elongated meshes more or less straight, wide and a majority are long. Isomorphic meshes are observed from elytral apex to ½ or ¾ of its length and at the anterior parts of the suture.
F	Longitudinally elongated meshes more or less straight to ½ of elytral length after that the meshes bend out towards suture ¼ from apex. Isomorphic meshes at the elytral apex and along anterior parts of the suture.
G	Isomorphic meshes cover the whole elytra, giving an impression that the longitudinally elongated meshes bend out towards suture ¼ from apex.

Seven categories (A-G) ranging from tightly packed longitudinally elongated meshes to more or less isomorphic meshes were recognised in *Agabus bipustulatus*. Type A is found in the *bipustulatus* and *solieri* forms, type B in *kiesenwetterii*, *falcozi* and *dolomitanus*, and type C, F and G in *pyrenaeus*. The categories D, E and F describe intermixed patterns.

The Simpson index (D), which measures the probability that two individuals randomly selected from a sample will belong to the same reticulation category, was used to calculate reticulation diversity per population [33].

### Genetic Analysis

A total of eleven enzyme systems coding for nine loci gave quantitatively reliable results: Hexokinase (*Hk*), Triosephosphate isomerase (*Tpi*), α-Glycerophosphate dehydrogenase (α*-Gpdh*), Isocitrate dehydrogenase (*Idh*), Malate dehydrogenase (*Mdh*), Malic enzyme (*Me*) and Esterase (*Est-1* & *Est-2*). In addition, Alkaline phosphate (*Alp*) was screened in *A. bipustulatus* from France and Italy, and so were Phosphoglucoisomerase (*Pgi*), Phosphogluconate dehydrogenase (*Pgd*) and Xanthine dehydrogenase (*Xdh*) in *A. bipustulatus* and *A. nevadensis* from Spain. Staining recipes and gel buffers are modified from those of Shaw and Prasad [34]. The beetles were prepared and the alleles named by migration distance to the most common allele as described in Drotz *et al.* [27].

Population structure was calculated as described by Weir and Cockerham [35] and genotypic linkage disequilibrium was tested for between loci per population. To evaluate if reticulation patterns influence population differentiation as assumed by sexual conflict in combination with assortative mating [36]–[37], we tested for differences in gene diversity, observed heterozygosity and *F*_st_ among groups consisting of specimens within populations with mixed and homogeneous reticulation patterns. Populations were grouped according to their variability in secondary reticulation observed as described above. Significant levels were calculated via a permutation scheme of 5000 replicates where the total sample is allocated at random to groups. The P-value is based on a two-tailed probability test of the proportion of the randomised data sets that gives larger mean values than the observed. All analyses were conducted in the FSTAT v2.91 software [38]. Pearson's correlation coefficient was estimated between altitude and mean heterozygosity and *F*_is_.

### DNA Amplification and Sequencing

DNA was extracted from one hind leg or the thoracic flight muscles of the frozen or alcohol preserved beetles, with a Qiagen DNeasy protocol for animal tissues. Partial cytochrome b (*Cyt b*) sequences were amplified with the two flanking primers CB-J-10933 and CB-N-11367, partial cytochrome c oxidase subunit I (*COI*) with CI-J-2195 and TL2-N-3014, and cytochrome oxidase II (*COII*) with C2-J-3279 and C2-N-3661 as described in Simon *et al.* [39] using the following polymerase chain reaction (PCR) program: denaturation 94°C (90 s), annealing at 50°C (30 s) and extension at 72°C (60 s). This cycle was repeated 30 times, followed by an extension period of 5 minutes. The PCR products obtained were single clear bands with no signs of non-specific amplification. The amplified product was approximately 430 bp in length for *Cyt b*, 730 bp for *COI*, and 400 bp for *COII*. The product was run on a 1.0 % agarose gel and then removed from the gel and purified with a Jetsorb DNA extraction kit. Sequencing reactions were performed with the DYEnamic ET terminator kit. Each sequence was sequenced from both its 3′ and 5′ ends. Corresponding accession number for the three partial mtDNA genes are AF439354-55, AF439359, AF439362, AF439365, AF439367-68, AF439372-77, and AY535285-400.

### Phylogenetic Analysis

The mtDNA sequences were aligned with the ClustalW multiple alignment option in BioEdit, version 4.8.10 [40]. No gaps were inserted within the alignment. Congruence among data sets was tested with the partition homogeneity test [41]. Unweighted parsimony analysis was performed by applying heuristic search with tree bisection-reconnection branch swapping. A total of 3000 searches were done with 100 replicates and ten random-addition sequence iterations per search started from a random tree. Branches were collapsed if branch length was minimum zero. All characters were nonadditive, and uninformative characters were excluded before the analysis. To evaluate if the data set is subjected to ‘long branch attraction’ we compared the strict consensus tree topology between two phylogenetic analysis; the first including all sequences from all outgroups and the second analysis including only the sequences from the ingroup as described by Bergsten [42]. The second dataset was also analysed with a maximum likelihood analysis. The Akaike Information Criterion in Modeltest [43] was used to find, the best evolutionary model given the data. Cladograms were rooted between the ingroup and outgroup according to Nixon and Carpenter [44]. Nodal support within the phylogenetic trees within the parsimony analysis was assessed with bootstrap percentage after 1000 re-sampling steps [45] and within the maximum likelihood analysis with jackknife percentage after 1000 re-sampling steps with 30% character deletion [46]. The number of extra steps required to collapse each clade was also calculated as described by Bremer [47]. Measures of how well all individual character fit on a phylogenetic tree is measured by the consistency index (CI). The average value is calculated by dividing the minimum possible number of steps by the observed number of steps on the tree. The retention index (RI) measures the amount of synapomorphy expected from a data set that is retained as synapomorphy on a tree. The above analyses were run in both PAUP v.4.0b10 [48] and WinClada ver. 0.9.99 [49]. Tajima's Neutrality test for the mtDNA sequence data [50] was performed with DNAsp [51] and the assumption of a local or global molecular clock was tested with PHYHYP v. 1 [52]. The Kishino and Hasegawa [53] test was used to test for two competing trees of equal length. To evaluate differences between tree topologies of unequal length we performed a permutation test where character states are randomly reassigned within characters of all taxa. Length differences between all permutated trees generate a null distribution that is used to compare the observed length differences of the original topologies. Significant level is gained in both analyses from a two-tailed probability test in PAUP v.4.0b10 [48].

Reticulation patterns were not optimised as a character on any of the parsimonious fundamental trees. Instead we here utilized the strict consensus to study the within and between clade distribution of reticulation patterns over all parsimonious fundamental trees. This method is well suited to distinguish if reticulation patterns have evolved multiple times within different monophyletic clades [23].

## Results

### Reticulation Diversity

**Figure 3:**
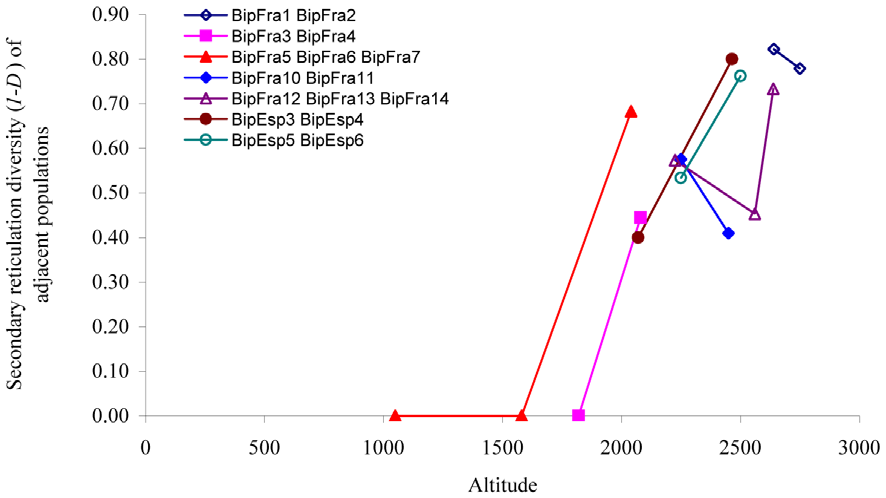
Relationship between male secondary elytral reticulation diversity and altitude in *Agabus bipustulatus*. Reticulation diversity was estimated with the Simpson diversity index (*D*) based on the data given in table 3. Low index value (1-*D*) indicates small level of variation in the secondary reticulation. Geographically close populations are connected with lines. Altitude is given in meters. Locality codes follow table 1.

The secondary reticulation varies noticeably within almost all populations from France, Italy and Spain (Table 3). In three populations (BipFra4, 6 and 7), all at an altitude below 2000 m a.s.l near Grenoble, all specimens of the same sex had similar reticulation patterns. The other populations, sampled at higher altitudes, consisted of a mix of A and B patterns in various frequencies with one or several individuals representing the D, E or F patterns. The exceptional Spanish population BipEsp5 did not have any males of neither the A nor B type, and instead we here found the C, F and G patterns dominating in both sexes (Table 3). Some geographically very close populations, separated by altitude, as BipFra5 and BipFra6 or BipFra13 and BipFra14, had different dominating male elytral patterns (A and B, respectively). In males the variation is significantly correlated with altitude (p<0.001, adj-R^2^ = 60.2%, b = 0.79). Increased diversity of reticulation patterns is seen in several independently parallel areas in Spain and France (Fig. 3). In females, a similar pattern, however not significant, of high diversity at high altitude is also observed.

**Table 3:** *Agabus bipustulatus* population variation of secondary elytral reticulation.

Locality code	Alt	Males									Females								
			A	B	C	D	E	F	G	*N*		A	B	C	D	E	F	G	*N*
BipFra1	2750		2	5	1	3	1	8		20		17	2			1	1		21
BipFra2	2640		4	6		2	4	7	1	24		20			1	2	1		25
BipFra3	2080		14	3				2		18		13							13
BipFra4	1820		13							13		14							14
BipFra5	2040		6	15			3	6		30		6	9			2	1		18
BipFra6	1049		30							30		28							28
BipFra7	1580		5							5		8							8
BipFra8	2080		21	4						25		27							27
BipFra9	2240		7	1				1		11		16							16
BipFra10	2250		5	17		1	1	3		28		30							30
BipFra11	2450		2	20	1		1	2		26		22	3						25
BipFra12	2560		2	22			1	4	1	30		30							30
BipFra13	2638			4		1	4		1	5		19							19
BipFra14	2225		14	9				2		25		19							19
BipIta1	2207		2	1		1			2	6		5							5
BipEsp3	2070		4	1						5		8							8
BipEsp4	2465		1	2	2					5		4							4
BipEsp5	2500				2			3	2	7			4	6				5	15
BipEsp6	2250		6	4						10									0
BipEsp7	2440		1							1		6		1					7

Male and female variation is given separately in different columns. Total number of specimens (*N*), altitude (Alt) is given in meters above sea level, and locality codes follow table 1. Elytral reticulation codes follow table 2.

### Genetic Variability

The within population variation in reticulation pattern made it difficult to assign a population into a given form. Populations were therefore divided into four groups based on the most frequent reticulation pattern in order to test for genetic differentiation between groups: (1) type A in all females and males (BipFra4, 6 and 7); (2) type A in all females and >50% type A in males (BipFra3, 8, 9 and 14); (3) type A in all females and >50% type B in males (BipFra9, 10, 12 and 13); (4) both sexes with variable reticulation patterns (BipFra1, 2, 5 and 11).

**Figure 4:**
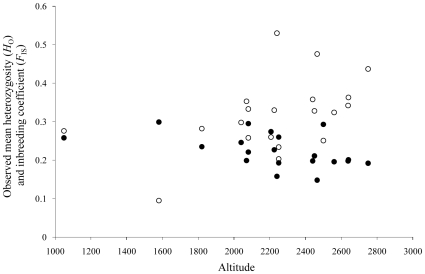
Relationship between observed mean heterozygosity and estimated inbreeding over populations, and altitude in *Agabus bipustulatus*. Estimated (•) observed mean heterozygosity (*R^2^-adj* =  0.185, *P* =  0.033); (○) estimated inbreeding coefficient (*F*_IS_) over populations (*R^2^-adj* =  0.168, *P* =  0.041). Altitude is given in meters.

Population mean heterozygosity and the mean number of alleles per locus, within the total sample (BipFra1-14, BipIta1 and BipEsp3-7) of *A. bipustulatus*, ranged between 0.158–0.299 and 1.9 – 3.1, respectively in the French Alps populations, and between 0.148 – 0.293 and 1.8 – 2.3 in the Spanish populations. The single Italian *dolomitanus* population exhibits similar values to those observed in Spain (Table 4). In addition, the mean heterozygosity (*r* = -0477, p =  0.033, R^2^-adj = 18.5%) and inbreeding coefficient within population (*F*_IS_; *r* = 0.461, p =  0.041, R^2^-adj = 16.8%) were both significantly correlated to altitude (Fig. 4).

**Table 4:** Summary of genetic variability in *Agabus bipustulatus*.

Locality code	Mean ind./locus	Mean # of alleles / locus	*F*_is_	Mean heterozygosity ± SE
BipFra1	26±1	2.3±0.4	0.437	0.192±0.04
BipFra2	26±1	2.1±0.4	0.363	0.201±0.07
BipFra3	18±1	2.6±0.6	0.258	0.295±0.07
BipFra4	16±1	2.3±0.3	0.282	0.235±0.05
BipFra5	42±2	2.5±0.5	0.298	0.246±0.06
BipFra6	39±3	3.1±0.4	0.276	0.258±0.07
BipFra7	11±1	1.9±0.2	0.095	0.299±0.09
BipFra8	37±4	3.0±0.6	0.333	0.221±0.06
BipFra9	11±2	2.3±0.4	0.530	0.158±0.05
BipFra10	34±5	2.8±0.6	0.234	0.260±0.06
BipFra11	36±6	2.0±0.3	0.328	0.211±0.06
BipFra12	40±4	2.1±0.4	0.324	0.196±0.05
BipFra13	13±2	2.3±0.5	0.342	0.198±0.06
BipFra14	29±3	2.8±0.5	0.330	0.227±0.07
BipIta1	9±1	1.8±0.3	0.358	0.198±0.08
BipEsp3	13±1	2.1±0.2	0.260	0.274±0.04
BipEsp4	14±1	2.1±0.2	0.353	0.199±0.04
BipEsp5	22±1	2.1±0.2	0.476	0.148±0.04
BipEsp6	12±1	2.3±0.3	0.251	0.293±0.05
BipEsp7	6±1	1.8±0.2	0.203	0.193±0.08

Including mean number of successfully scored individuals per population over all loci, mean number of alleles per locus including ±1 standard error (SE), mean observed heterozygosity estimated as direct count including ±1 SE, and inbreeding coefficient per population measured by *F*_is_. Locality codes follow table 1.

No significant differences were detected between the above described reticulation groups in observed heterozygosity (p = 0.67), or genetic diversity (p = 0.44) or *F*_st_ (p = 0.45). Significant genotypic disequilibrium was only found between the *Idh* and *Mdh* loci in one French population (392 000 permutations, p-value 0.00001; adjusted *P*-value for 5% nominal level 0.000128).

*Agabus nevadensis* displays a significant deviating pattern in observed heterozygosity (p = 0.0054), genetic diversity (p = 0.0008) and allele frequencies in relation to *A. bipustulatus*. The mean heterozygosity of *A. nevadensis* is *H* = 0.134±0.044 and the mean number of alleles per locus is 1.9. Of the compared enzyme systems, the allele frequencies of *Hk* and *Pgd* stand out. The *Pgd* locus has three alleles in the Spanish *A. bipustulatus* sample, but only two in *A. nevadensis*. The allele that is missing in *A. nevadensis* is, interestingly, the most commonly found in *A. bipustulatus* in north Spain (*Pgd* ^97^), and the most commonly found allele in *A. nevadensis* (*Pgd* ^102^) is the least frequently found in *A. bipustulatus* in north Spain. A similar difference is seen in the allele frequency of the *Hk* locus. Throughout *A. bipustulatus* populations in Spain and France this locus consists of two alleles with a higher occurrence of allele *Hk* ^100^ in most populations, whereas in *A. nevadensis* this locus is more or less monomorphic for the *Hk* ^102^ allele in the eight studied populations. There is clearly a reproductive barrier between these two species.

### Sequence Variation

Combined, the three mtDNA data sets *COI*, *COII* and *Cyt b* included 1236 base pairs. A total of 214 sites were parsimony-informative over all taxa, 150 within the *A. tristis* group, and 33 within the *A. bipustulatus* complex. Of the 214 informative characters, 174 were from the third, 34 from the first, and 6 from the second position. Translation of nucleic acid to amino acid sequences revealed very few parsimony-informative characters; i.e. most of the variation seen represents silent mutations. The incongruence length difference test was not significant between any combination of the three data sets. Most of the character conflicts in the data are within the separate sets, and only 2.33–4.19 % increased homoplasy was found in the total data set including all taxa.

Tajima's test of the neutral mutation hypothesis of the *A. nevadensis* and the *A. bipustulatus* complex sequences showed no significant deviation (p>0.10) between the amount of segregating/polymorphic sites and the average number of nucleotide differences, number of segregating sites (S)  = 52, Theta per sequence = 13.16036 and 0.01066 per site, nucleotide diversity, Pi = 0.00826 and Tajima's D = −0.83312.

### Elytral Reticulation and Phylogeny

**Figure 5:**
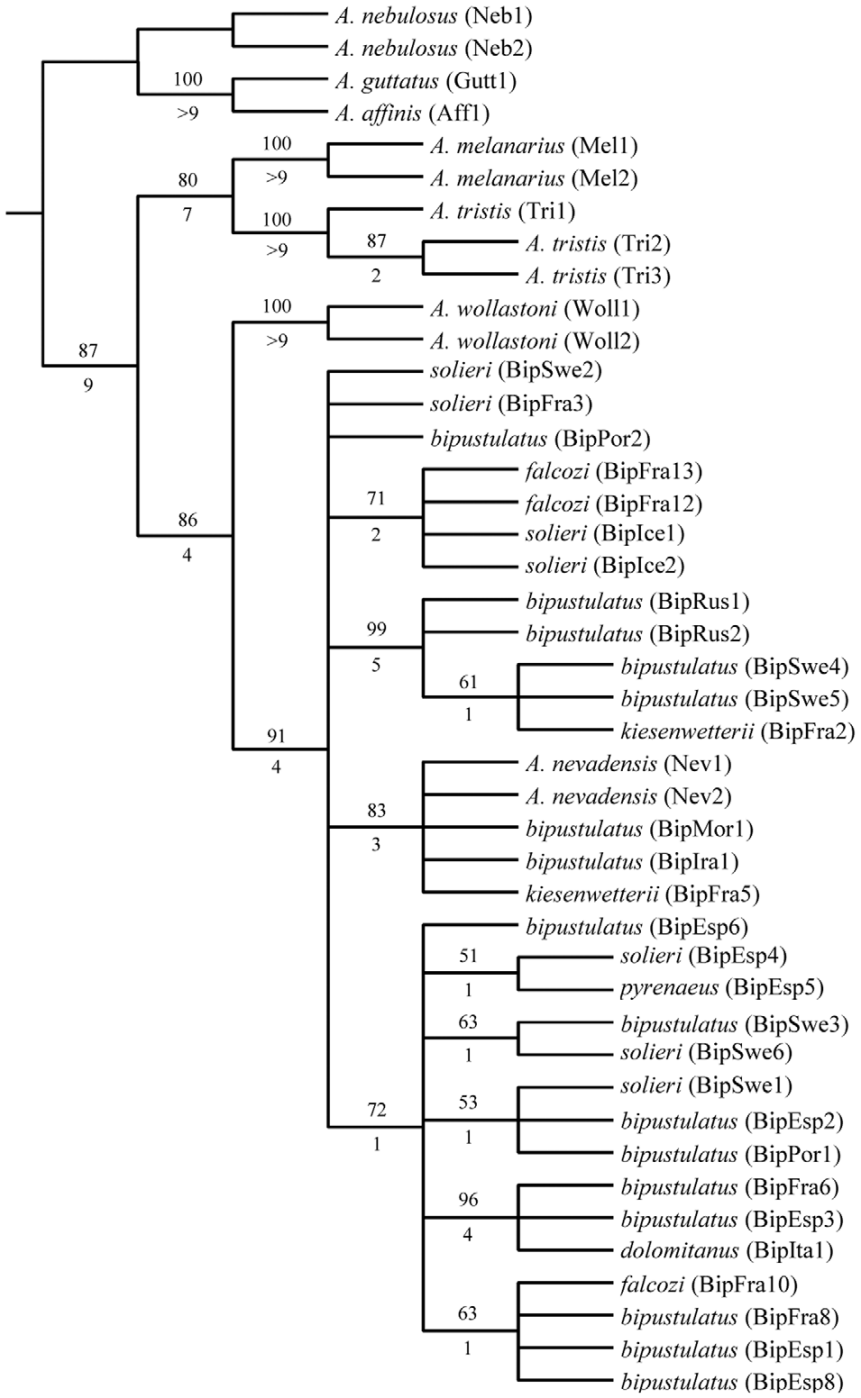
Strict consensus tree from parsimony analysis of *Agabus bipustulatus* complex plus outgroups. The combined unweighted parsimony analysis includes all three genes (cytochrome b, cytochrome c oxidase subunit I and cytochrome oxidase II) and the outgroup species *Agabus nebulosus, A. affinis* and *A. guttatus*. Number of fundamental trees = 13. Bootstrap values above 50% are reported above branches. Bremer support values are reported below branches. Locality codes follow table 1.

**Figure 6:**
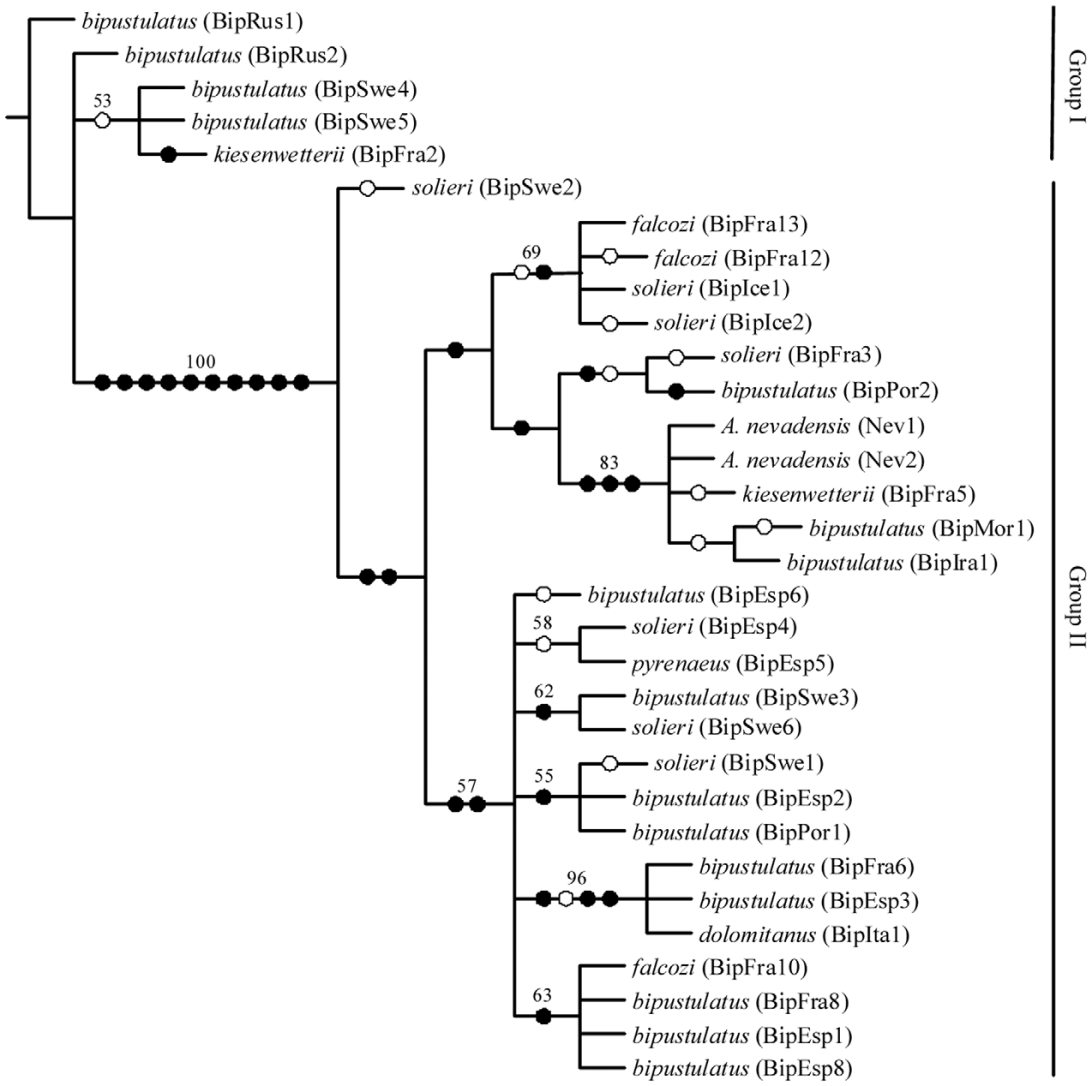
tree from parsimony analysis of *Agabus bipustulatus* complex. The “best” fundamental tree, according to Kishino and Hasegawa test, from combined unweighted parsimony analysis of all three genes (cytochrome b, cytochrome c oxidase subunit I and cytochrome oxidase II), rooted with *Agabus bipustulatus* specimen from BipRus1 in order to visualise the deep split within the complex (group I and II), and to display unambiguous unique character state transformations, marked with (•), and homoplasious character state transformations with (○). Bootstrap values above 50% are reported above branches.

Parsimony analysis of the combined data set, including all outgroup taxa, resulted in 13 most parsimonious (MP) trees with a length of 400 steps (uninformative characters excluded), and CI and RI of 0.65 and 0.83, respectively. These fundamental trees are combined into a strict consensus (Fig. 5). The subsequent phylogenetic analysis only including the *A. bipustulatus* complex and *A. nevadensis* specimens resulted in four MP trees with a tree length of 48 steps, CI and RI of 0.90 and 0.97, respectively. Main differences between these fundamental trees were the position of three haplotypes (BipSwe2, BipFra3 and BipPor2) and the grouping of the two clades consisting of the BipIce1, BipIce2, BipFra12, BipFra13 and the *A. nevadensis*, BipFra5, BipIra1, and BipMor1 specimens. The topology and subclades of the strict consensus tree from both analyses were identical, which provides strong evidence that no long branch attraction is affecting the results. The Kishino and Hasegawa test of the four MP fundamental trees of the *A. bipustulatus* complex resulted in one phylogenetic hypothesis with the lowest likelihood value. This tree is hereafter referred to as the “*best*” tree (Fig. 6).

**Figure 7:**
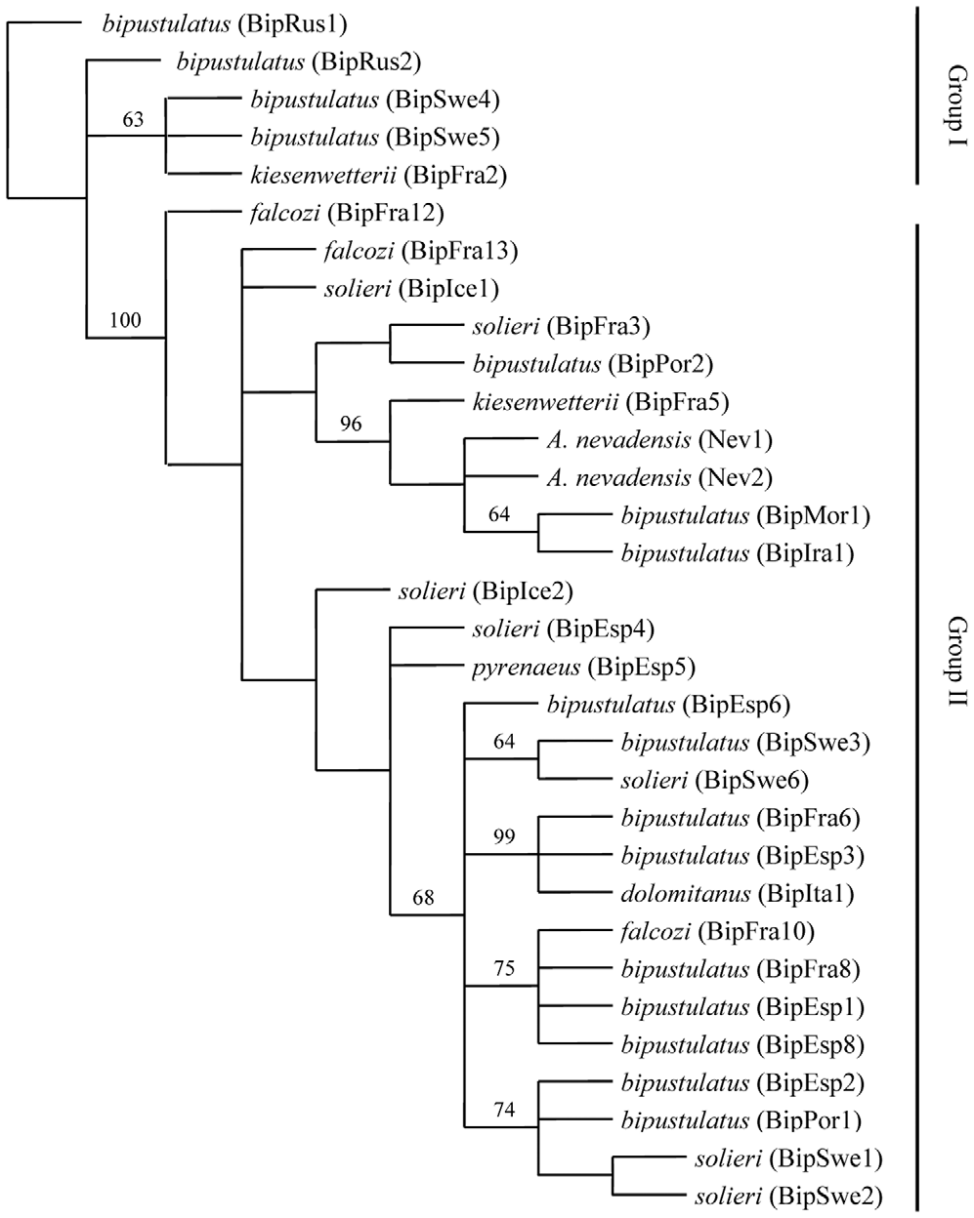
Maximum likelihood representation of sequence data from *Agabus bipustulatus* complex. Likelihood representation including all three genes (cytochrome b, cytochrome c oxidase subunit I and cytochrome oxidase II). The translational model (TIM) was used along with a proportion of invariable sites equal to 0.8199 and a gamma distribution shape parameter of 0.7877. The tree is rooted with the *Agabus bipustulatus* specimen from BipRus1 in order to visualise the deep split within the complex (group I and II). Jackknife values above 50% are reported above branches.

According to the Akaike Information Criterion in Modeltest, the Translational model (TIM), with a proportion of invariable sites equal to 0.8199 and a gamma distribution shape parameter of 0.7877 is the best evolutionary model, within the maximum likelihood analysis, given the data only including the *A. bipustulatus* complex and *A. nevadensis* specimens (-lnL 2954.8911 and AIC score 4125.7822). Nucleotide frequencies; (A) 0.3368, (C) 0.1501, (G) 0.1214 and (T) 0.3917 and evolutionary model parameters; A-C: 1.0000, A-G: 11.6809, A-T: 0.1441, C-G: 0.1441, C-T: 6.3908 and G-T: 1.0000. The maximum likelihood analysis resulted in six trees after 75166 rearrangements tried from a starting Neigbour Joining tree. From these trees the Kishino-Hasegawa (KH) test selected significantly one tree (ln 348.43962) to be the most likely representing the evolutionary history of the data (Fig. 7).

Two main evolutionary lineages are present within the “best” MP tree (Fig. 6) and the ML tree (Fig. 7), hereafter referred to as group I and II. These two groups are separated by 10 unambiguous unique nucleotide transformations, which result in both a 100% bootstrap (BS) and Jackknife support (JS). Group I includes specimens from two haplotypes from Russia, two from Sweden and one from France, whereas all other specimens from Sweden, France, Spain, Island, Italy, Morocco, and Iran belong to group II. The “best” MP tree (Fig. 6) and the ML tree (Fig. 7) differ chiefly in the position of the BipSwe2 haplotype and the presence or absence of the subclade including the haplotypes BipIce1, BipIce2, BipFra12 and BipFra13, and the subclade including BipEsp4 and BipEsp5. These differences do not change the evolutionary interpretation of the morphological results of this study, however. Both kinds of phylogenetic analyses of the *A. bipustulatus* complex clearly demonstrate a multiple independent origin of different reticulation patterns. This pattern is evident in the *kiesenwetterii* form (B type) that is found in several of the monophyletic groups in the MP strict consensus tree together with haplotypes of the *bipustulatus* form (A type) (Fig. 5 and Fig. 7). The *falcozi* form also displays a similar pattern and is found within two well-supported subclades (71 and 63% BS, respectively) within the MP analysis, and basally of group II and in one of its subclades in the ML (75% JS). The *dolomitanus* form (B type) from Italy (BipIta1) forms a strongly supported subclade (96% BS and 99 % JS) together with the *bipustulatus* form (A type) from France (BipFra6) and Spain (BipEsp3). Moreover, the grouping of *bipustulatus*, *solieri* and *kiesenwetterii* as sister forms within several well supported subclades (BS and JS 53–93%) strengthens the argument of adaptation to altitudinal environments.

Forcing the *kiesenwetterii*, *falcozi* or *solieri* forms on the “best” MP fundamental tree (Fig. 6) to be monophyletic resulted in a dramatic increase in three length of 71.1 % compared to the fundamental trees. Still an even larger increase (83.3%) is seen when we add an additional topological constraint assuming *A. nevadensis* as sistergroup.

*Agabus nevadensis* is deeply nested in group II, within the *A. bipustulatus* complex (Fig. 5 and Fig. 7). However, the allozyme differences seen in the population genetic analysis imply that the adaptation process can lead to assortative mating and the evolution of a reproductive barrier.

## Discussion

We have studied the phylogeographic history and genetic variation of different reticulation patterns across the *A. bipustulatus* complex and *A. nevadensis* distribution in the West Paleartic in order to understand the complex variation in elytral reticulation that occurs in both sexes at high altitudes in Central and South Europe in contrast to the more homogeneous reticulation pattern normally observed at lower altitudes across the total distribution area.

We found that the different secondary reticulation patterns in *A. bipustulatus* have evolved independently at several times. The phylogenetic pattern shown here within the “best” MP fundamental and ML tree describes the *A. bipustulatus* complex as a deep gene tree with two major lineages broadly sympatric, between specimens with different reticulation patterns (Fig. 6). This reflects a species with a large evolutionary effective population size and high gene flow, with a recent admixture of lineages that have diverged allopatrically [54]. The geographic mix of sampled specimens within the recognised monophyletic group I and II, of *A. bipustulatus* indicates that the Pyrenees and Alps have not acted as dispersal barriers in the interglacial periods, which is argued to be a major potential isolation barrier for the Iberian water beetles [31]. Instead specimens of *A. bipustulatus* have been able to disperse more or less randomly into different areas during the climatic oscillations that have occurred during the late Pleistocene [55]. In addition, specimens from both groups I and II co-occur at the Stintbäcken locality in north Sweden. There is therefore no reason to believe that the deep gene tree represents two different monophyletic genetically isolated entities, since the genetic structure of the Stintbäcken population does not differ from other *A. bipustulatus* populations across its distribution area [56].

Our results also indicate the presence of different selective regimes at different altitudes. The lack of significant genetic differences among *A. bipustulatus* populations representing combinations of reticulation patterns indicates that the historical legacy of a population does not hinder gene flow [57]–[58]. However, decreasing mean heterozygosity and increased population substructure (*F*_IS_) at different altitudes regardless of reticulation patterns shows that the selective process, is strong enough to alter/change the genetic structure of populations (Fig. 4 and Table 4). This genetic pattern is congruent with earlier findings in north Scandinavia [27], [56]. Individual response in secondary reticulation at high altitude is observed as widening of the longitudinally elongated secondary meshes and increased coverage of isomorphic meshes from the apex up to half of the elytral length. Males are more affected than females (Table 3). The occurrence of especially secondary reticulation type C and G specimens in France and Italy at very low frequencies together with other reticulation patterns outside the Vall de Boí in Catalunya (Table 3) implies that crosses between reticulation patterns may give rise to patterns not observed within parents seen as increased reticulation diversity at higher altitude. The geographical and altitudinal distribution of the reticulation types and the genetic variation within the *bipustulatus*-complex suggest a complex evolution. The enlarged isomorphic meshes of the secondary reticulation at high altitude in females and the evolution of wider male tarsi and attached suckers in the presence of modified reticulated females could indeed contribute to the arms-race between sexes proposed by Howe [13]. An alternative explanation for the increased variance in female reticulation at high altitude could be that it arises as a side effect of the greater variation of elytral reticulation in males in these populations.

The high altitudinal populations of *A. nevadensis* stand out in comparison to the genetic variation found among populations of *A. bipustulatus* and their phylogenetic position deeply nested within the *A. bipustulatus* group II (Fig. 6 and 7). The large similarity of the primary and secondary reticulation in *A. nevadensis* to that of the *kiesenwetterii* form (type B, Table 2) suggests that the initial altitudinal adaptation follows the same selection process as the other reticulation patterns in *A. bipustulatus* (type A). However, the significant differences in allele frequencies between *A. nevadensis* in Sierra Nevada and *A. bipustulatus* populations in Spain, France, Italy and Sweden at different altitudes strongly indicate that *A. nevadensis* has relatively recently became reproductively isolated *in situ* [59]–[61].

Drotz *et al.* [27] documented that selection acted on α-Glycerophosphate dehydrogenase (α*-Gpdh*) in *A. bipustulatus* between the valley floor and above the tree line in Scandinavia. This selective force is, however, not responsible for the observed reticulation variation seen in both *A. nevadensis* and *A. bipustulatus* since it occurs across the species total distribution area and affect all analysed forms of both species regarding their reticulation pattern [56]. Studies of the leaf beetle *Chrysomela lapponica* could, however, give some clue to a possible selective candidate. This species displays a patchy distribution of elytral colour variation with large dark black spots in northern Europe and high mountainous regions in Western Europe. In central Europe, brightly red coloured beetles are more common at low altitude. Gross *et al.* [62] demonstrated empirically that the increase in melanic elytra in northern Europe and high altitude represent a selective thermal advantage at low temperatures in relation to the brightly coloured central European form. Here the size of the dark black spots regulates the body temperature. Similar demographic distribution and colour morph variation is seen in *Coelophora inaequalis, Harmonia axyridis* and *Oreina sulcata* [63]–[65]. The colour variation within these species is assumed to be driven by solar UV-*β* radiation and interestingly solar UV-*β* radiation normally increases with increasing altitude. This implies that the strongest selective effect within a geographical area is overlapping with our observed deviation in reticulation pattern of *A. bipustulatus* and *A. nevadensis*. The Alps, Pyrenees and Sierra Nevada display the highest UV levels in Europe as a consequence of their high altitude, snow-covered surface and low aerosol levels [66]. In addition, the effect of UV radiation in aquatic environments is largest in shallow mountain lakes with high incident flux and deep penetration of UV radiation [67]. This habitat is often associated with *A. bipustulatus* species at high altitude [68]. Here different reticulation patterns may reflect UV radiation differently where more impressed primary reticulation can lead to increased body temperature [69].

Further research is needed in order to fully understand the interplay between the selective forces that clearly plays a part in forming the reticulation pattern observed within the *A. bipustulatus* complex and their combined importance for adaptive/sympatric speciation. One way to do this could be to address how the relative strength of sexual and natural selection changes at different altitudes, by analysing male reticulation and suction cup variation in relation to the female reticulation patterns at different altitudes. This, however, is beyond the scope of this paper.
